# Optimizing Sleep in Older Adults: Where Does High-Intensity Interval Training Fit?

**DOI:** 10.3389/fpsyg.2020.576316

**Published:** 2020-10-21

**Authors:** Alexis Bullock, Ana Kovacevic, Tara Kuhn, Jennifer J. Heisz

**Affiliations:** Department of Kinesiology, McMaster University, Hamilton, ON, Canada

**Keywords:** aging, exercise, sleep quality, sleep efficiency, physical health, mental health

## Abstract

The present community-based study evaluated the effect of three different exercise interventions on sleep quality. Older adults were enrolled in one of three exercise intervention groups: high-intensity interval training (HIIT; *n* = 20), moderate-intensity continuous training (MICT; *n* = 19) or stretching (STRETCH; *n* = 22). Prior to and following the intervention, sleep quality was assessed using the Pittsburgh Sleep Quality Index (PSQI). The PSQI was used to classify participants as poor (global PSQI score ≥5) or good (global PSQI score >5) sleepers and the effect of the intervention was examined on poor sleepers only. Around 70% of our sample was classified as poor sleepers. Poor sleepers were significantly impaired across all PSQI components, except for the use of sleeping medication, such that neither group was heavily prescribed. Exercise improved sleep quality for poor sleepers, but the intensity mattered. Specifically, MICT and STRETCH improved sleep efficiency for poor sleepers, whereas HIIT did not (*p* < 0.05). The results suggest that both MICT and STRETCH may be more effective than HIIT for optimizing sleep in poor sleepers. These findings help to inform exercise guidelines for enhancing sleep in the aging population.

## Introduction

Sleep is vital for optimal health (Reid et al., 2006), but its quality declines with age (Espiritu, 2008) and this increases the risk of morbidity and mortality (Henderson et al., 1995; Foley et al., 1999; Dew et al., 2003). Aerobic exercise is a potential strategy for improving sleep quality in older adults (Youngstedt, 2005; Yang et al., 2012; Kredlow et al., 2015; Bonardi et al., 2016), especially for those with pre-existing sleep impairments (i.e., poor sleepers; King et al., 1997, 2008; Tworoger et al., 2003; Passos et al., 2011; Yang et al., 2012). However, it remains unclear whether all types of exercise confer similar benefits on sleep.

Much of the research in this field has focused on moderate-intensity continuous training (MICT), demonstrating beneficial effects on both objective and subjective measures of sleep quality (King et al., 1997, 2008; Tworoger et al., 2003; Passos et al., 2011; Yang et al., 2012). With respect to objective measures, a 12-month moderate-intensity aerobic exercise intervention with older adults found significant changes in sleep architecture compared to controls, with less time spent in Stage 1 sleep, more time in Stage 2 sleep, and overall fewer awakenings during the first third of the sleep period (King et al., 2008). With respect to subjective measures, older adults report improvements on various aspects of the Pittsburgh Sleep Quality Index (PSQI) following MICT, including the global PSQI sleep score, sleep quality, sleep onset latency, sleep disturbance, and sleep duration (King et al., 1997, 2008). Thus, there is accumulating evidence that MICT improves sleep in older adults, but more work is needed to investigate other modalities of exercise and refine the optimal exercise prescription for promoting sleep in the aging population.

High-intensity interval training (HIIT) is a popular alternative to traditional MICT in young and older adults. Compared to MICT, HIIT produces similar-to-superior improvements in physiology and cognition in older adults (Wisløff et al., 2007; Coetsee and Terblanche, 2017), with the added benefit of requiring less time. With respect to sleep outcomes, an *acute* bout of high-intensity exercise may be equally effective at optimizing sleep as moderate intensity exercise (Kredlow et al., 2015). However, a recent meta-analysis was unable to examine the effects of *chronic* high-intensity exercise training on sleep due to insufficient evidence (Kredlow et al., 2015). To our knowledge there has only been one study investigating the effect of HIIT on sleep quality (Adams et al., 2018). This was a 12-week exercise intervention conducted with testicular cancer survivors and found no improvements in sleep quality (Adams et al., 2018). More work is clearly needed to understand whether a regular program of HIIT provides similar or greater benefits compared to MICT for sleep promotion.

In addition to aerobic exercise, stretching has been considered a potential strategy for improving sleep in older adults (King et al., 1997; Morin et al., 1999; Tworoger et al., 2003). One study found that both aerobic exercise and stretching improved sleep quality in postmenopausal women (Tworoger et al., 2003). Although most research examining the effects of exercise on brain health use stretching as a control, in the context of sleep, stretching does not function as a true control because of its potential to influence sleep, possibly through physical relaxation (King et al., 1997), and thus represents another viable modality of exercise for improving sleep in older adults.

The current study examined the effects of exercise type on sleep quality in older adults. This study used archival data from a community-based study that examined the role of exercise intensity on cognition in older adults (Kovacevic et al., 2019). Sedentary but otherwise healthy older adults over the age of 60 years were recruited to participate in this study. Participants were assigned to one of three intervention groups: HIIT, MICT, or a stretching (STRETCH) group. Sleep quality was assessed using the PSQI. We hypothesized that MICT would be effective at improving sleep quality but were unclear whether HIIT or STRETCH would confer similar benefits. As a secondary analysis, we split participants into poor and good sleepers using the PSQI, with particular interest in identifying interventions that benefit the poor sleepers. The impact of the interventions on cardiorespiratory fitness, body mass index (BMI), as well as measures of psychological stress, and global cognition were also examined.

## Materials and Methods

### Participants

This community-based study took place over a period of 2.5 years from August 2014 to March 2017. The methodology was reported by Kovacevic et al. (2019). Participants were recruited on a rolling basis throughout the study period through local news outlets and postings. Participants consisted of sedentary, but otherwise healthy community-dwelling older adults over the age of 60 years. Participants were excluded from the study if they reported engaging in more than 1 h of vigorous physical activity per week or if they had a known diagnosis of cognitive impairment. Participant eligibility was assessed through verbal or written confirmation *via* phone or email. Participants were also required to complete a stress test with their physician prior to enrolment to screen for any abnormal response to exercise. Those with abnormal responses to exercise were deemed ineligible. Eligible participants provided written informed consent and received $40 for participation upon study completion. This study received ethics clearance from the Hamilton Integrated Research Ethics Board.

### Procedure

The procedure consisted of pre and post-intervention assessments, separated by a 12-week intervention. The pre and post-intervention assessments included measures of sleep quality, as well as physical health, stress, and cognition. Post-intervention testing was completed within 48 h of the final intervention exposure.

**Figure 1:**
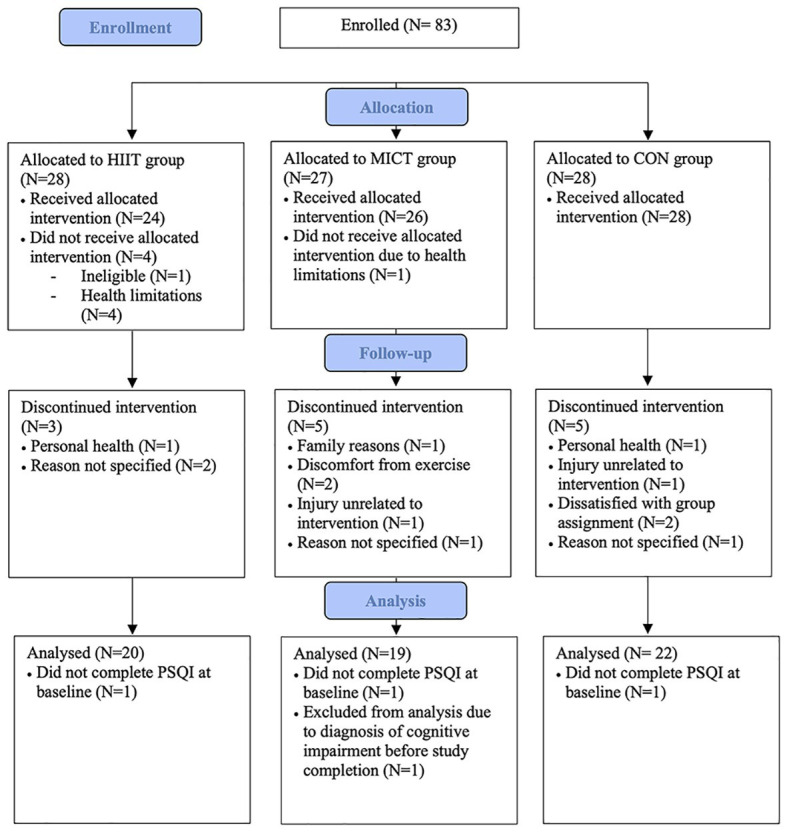
Flow of participants through study (Kovacevic et al., 2019).

Figure 1 displays the flow of participants through the study. A total of 83 participants enrolled in the study. Of those 83 participants, five participants withdrew before beginning training (HIIT: *n* = 4, MICT: *n* = 1) and 13 participants discontinued the intervention (HIIT: *n* = 3, MICT: *n* = 5, STRETCH: *n* = 5). A total of 65 participants completed the intervention. However, one participant in the MICT group was excluded from analysis due to a diagnosis of cognitive impairment prior to study completion and three participants did not complete the PSQI at baseline (HIIT: *n* = 1, MICT: *n* = 1, STRETCH: *n* = 1). Thus, the current sample is comprised of 61 participants. Participants trained in one of the three groups for the duration of the intervention: (1) HIIT (*n* = 20), (2) MICT (*n* = 19), or (3) STRETCH (*n* = 22). Group assignment was performed by a researcher and was done according to blocks stratified by sex to ensure equal distribution among groups. Group sizes were limited due to equipment availability, thus in the event that a group was full participants were assigned to the next available group. Group assignment took place prior to the pre-intervention assessment, but participants were not informed of their group placement until pre-intervention assessments were completed.

### Pre and Post-intervention Assessments

#### Sleep

Participants completed the 19-item PSQI to assess subjective sleep quality during the last month (Buysse et al., 1989). The primary measures of sleep quality derived from the PSQI were sleep duration and sleep efficiency. Data were screened to ensure sleep efficiency did not exceed 100%. Sleep duration was defined as the hours of sleep per night. Sleep efficiency was calculated using the following formula:

$$Sleep\;efficiency=\left(\frac{number\;of\;hours\;slept}{number\;of\;hours\;spent\;in\;bed}\right)\times 100$$

The 19 items on the PSQI were also combined to create seven component scores (range 0–3): (1) subjective sleep quality, (2) sleep latency, (3) sleep duration, (4) habitual sleep efficiency, (5) sleep disturbances, (6) use of sleeping medication, and (7) daytime dysfunction. The sum of the seven component scores yields a global score of sleep quality (range 0–21). Higher scores indicate worse sleep, with a cut-off score of 5 indicating clinical sleep impairment (Buysse et al., 1989).

#### Psychological Stress

The Perceived Stress Scale (PSS) was used to assess the participants’ perception of their psychological stress during the last month. This 10-item questionnaire measures the degree to which situations are considered stressful, where a higher score on the PSS is indicative of greater perceived stress (Cohen et al., 1983).

#### Global Cognition

The Montreal Cognitive Assessment (MoCA) was used to assess global cognition. The maximum score on the MoCA is 30, with higher scores indicating better cognitive function (Nasreddine et al., 2005).

#### Cardiorespiratory Fitness

Participants completed the modified Bruce protocol on a motor driven treadmill (Life Fitness 95Ti), with stages of 3 min in duration, as a predictive test of peak oxygen uptake (VO_2_ peak). The modified Bruce protocol introduced an adaptation phase of 6 min at the beginning of the test (Willemsen et al., 2010), while the remainder of the assessment followed the standard Bruce protocol (Sheffield and Roitman, 1976; Willemsen et al., 2010). A trained member of the research team supervised the test and recorded time at exhaustion, heart rate (HR) at each interval and at exhaustion (measured using Polar FT1 HR monitors), and rating of perceived exertion (RPE) at each interval using the Borg 6-to-20 scale (Borg, 1982). The test was terminated upon volitional exhaustion or presentation of abnormal symptoms. Predicted VO_2_ peak was calculated using the equation below, with a weighting factor of one for men and two for women (Bruce et al., 1973). The 6-min adaptation phase from the modified Bruce protocol was not included in this calculation.

$$\begin{array}{l} Predicted\;VO_{2}\;peak=6.70-2.82\left(weighting\;factor\;for\;sex\right)\\ \;\;\;\;\;\;+0.056\left(duration\;in\;seconds\right) \end{array}$$

Prior to completing the cardiorespiratory fitness assessment, participants were familiarized with the treadmill and completed the first 9.5 min (the first 3 stages) of the modified Bruce protocol, unless volitional exhaustion was reached earlier. Midway through training, an additional cardiorespiratory fitness assessment was completed to reassess peak heart rate (HR peak) and increase the difficulty of exercise training accordingly.

The first 10 participants that enrolled in the study completed the Single Stage Treadmill Walking Test (Ebbeling et al., 1990), instead of the modified Bruce protocol, as the pre-intervention cardiorespiratory fitness assessment. The modified Bruce protocol was subsequently used for all participants upon ethics approval. Participants who completed the Single Stage Treadmill Walking Test were excluded from analysis of cardiorespiratory fitness.

### Exercise Intervention

All groups met three times per week for the duration of the 12-week intervention and were supervised by a trained member of the research team. The 18th session was replaced with the additional cardiorespiratory fitness assessment, resulting in a total of 35 training sessions (actual completed: HIIT: 34 ± 4; MICT: 32 ± 5; STRETCH: 35 ± 2; M ± SD). Participants were instructed not to engage in additional physical activity for the duration of the study. Participants were accommodated for missed exercise sessions and continued to meet with their respective exercise groups until they achieved as close to the target of 35 training sessions as possible.

The HIIT and MICT protocols were adapted from previous work that was conducted in a sample of older adults with heart failure and thus was deemed feasible for our sample (Wisløff et al., 2007). The exercise protocols were designed to be matched for total training load and consequently their durations differed. All exercise training was completed on a motor-driven treadmill (Life Fitness 95Ti). The speed and incline of the treadmill were adjusted to elicit the target HR, which was based on the participant’s HR peak achieved during the fitness assessment, and target RPE. If both target HR and target RPE were not achieved simultaneously, HR was the preferred indicator of intensity.

#### High-Intensity Interval Training

The total time was 43 min. Participants warmed up for 3 min at 0% grade and 50–70% HR peak. Participants then completed 10 min at 5% grade and 60–70% HR peak. Next, they began the interval protocol, which consisted of four 4-min intervals at 5% grade and 90–95% HR peak, interspersed with 3-min of active recovery at 50–70% HR peak. After that, participants cooled down for 2 min at 50–70% HR peak. HR and RPE were recorded at the end of the warm-up, as well as after each 4-min interval and 3-min of active recovery. On average across each session, the HIIT protocol elicited a HR of 125 ± 9 beats/min, corresponding to 88 ± 4% of HR peak, and an RPE of 13.1 ± 1.7 out of 20 (M ± SD).

#### Moderate-Intensity Continuous Training

The total time was 52 min. For the first 3 min, participants warmed up by completing 3 min at 0% grade and 50–70% HR peak. For the next 47 min, they walked continuously at 70–75% HR peak. After that, participants cooled down for 2 min at 50–70% HR peak. HR and RPE were recorded after the warm-up and every 7 min for the remainder of the session. On average across each session, the MICT protocol elicited a HR of 104 ± 12, corresponding to 75 ± 5% of HR peak, and an RPE of 9.2 ± 1.6 out of 20 (M ± SD).

#### Stretching

The original study investigating the effects of exercise intensity on cognition intended that the STRETCH group act as a control for the exercise treatment arms (Kovacevic et al., 2019). However, the present study treated STRETCH as an additional treatment group, given the evidence that stretching may improve sleep quality (Morin et al., 1999; Tworoger et al., 2003), possibly through relaxation (King et al., 1997). The STRETCH group completed a series of non-aerobic seated and standing stretches in a large classroom. The sessions were 30 min in duration. The stretching protocol was designed for older adults and aimed at whole-body stretching. Each stretch was held for approximately 30–40 s. RPE was recorded at the end of each session. On average, the STRETCH protocol elicited an RPE of 8.2 ± 1.7 out of 20 (M ± SD). HR was not recorded during the STRETCH protocol.

### Statistical Analysis

All data was analyzed using IBM SPSS Statistics Software 25. Data were checked for extreme outliers, which were defined as values greater or less than three standard deviations (SD) from the mean. Two outliers were identified (pre-intervention PSS: STRETCH = 1; global PSQI change score: HIIT = 1). Data were then screened for missing cells; 7% of the data were missing. Outliers and missing cells were excluded pairwise. Normality was assessed using the Shapiro-Wilk test and through visual inspection of histograms.

#### Baseline Characteristics: Group Effects

One-way analyses of variance (ANOVAs) were conducted to determine if there were any baseline differences between groups (HIIT, MICT, and STRETCH) in age, PSQI scores (global sleep quality, sleep efficiency, sleep duration, and components one through seven), VO_2_ peak, BMI, PSS, or MoCA. *Post hoc* pairwise comparisons were performed to examine group effects.

#### Exercise Intervention: Group Effects

To evaluate the effect of the exercise intervention on sleep quality, analyses of covariance (ANCOVAs) were conducted on PSQI change scores (post-pre) of global sleep quality, sleep efficiency, sleep duration, as well as components one through seven, with a between-subjects factor of group (HIIT, MICT, and STRETCH). Age, sex, and BMI have been reported to effect sleep quality (Redline et al., 2004; Lauderdale et al., 2009) and thus, were included as covariates in all sleep analyses. *Post hoc* pairwise comparisons were performed to examine group effects.

To evaluate the effect of the intervention on cardiorespiratory fitness, an ANCOVA was conducted on the change score (post-pre) of VO_2_ peak with a between-subjects factor of group (HIIT, MICT, and STRETCH). Age and sex were included as covariates. *Post hoc* pairwise comparisons were performed to examine group effects.

#### Baseline Characteristics: Subgroup Effects

As a secondary analysis, we used the measure of global sleep quality to identify poor sleepers (global PSQI score ≥5) and good sleepers (global PSQI score <5; Buysse et al., 1989; Irwin et al., 2008). To characterize the sample of poor and good sleepers at baseline, we conducted ANCOVAs with a between-subjects factor of sleeper status (poor sleeper, good sleeper) on PSQI scores (global sleep quality, sleep efficiency, sleep duration, and components one through seven), with age, sex, and BMI included as covariates, as well as on VO_2_ peak, BMI, PSS, and MoCA, with age and sex included as covariates.

#### Exercise Intervention: Subgroup Effects

Next, we evaluated the effect of the exercise intervention on sleep quality in poor sleepers only. From an intervention standpoint, poor sleepers are the target group for treatment. From a statistical standpoint, the random sample of older adults drawn for the original study examining cognition included only a small sample of good sleepers that was insufficient for comparison (HIIT: *n* = 5, MICT: *n* = 4, STRETCH: *n* = 9). For poor sleepers, ANCOVAs were conducted on PSQI change scores (post-pre) of global sleep quality, sleep efficiency, sleep duration, as well as components one through seven, with a between-subjects factor of group (HIIT, MICT, and STRETCH). Age, sex, and BMI were included as covariates. *Post hoc* pairwise comparisons were performed to examine group effects.

#### Cardiorespiratory Fitness and Sleep

To explore the relationship between cardiorespiratory fitness and sleep, we conducted partial correlations between VO_2_ peak change scores and change scores for any sleep variable significantly affected by the intervention. Age, sex, and BMI were controlled for.

For all ANOVAs and ANCOVAs, partial eta square effect sizes are reported and can be interpreted as small (0.01), medium (0.06), and large (0.14; Miles and Shevlin, 2001).

## Results

### Baseline Characteristics: Group Effects

Baseline characteristics are reported in Table 1. Prior to the intervention, there was a main effect of group on cardiorespiratory fitness [*F*(2, 47) = 4.50, *p* = 0.016, *η_p_*^2^ = 0.16], such that HIIT and MICT had significantly higher VO_2_ peak at baseline than STRETCH (HIIT vs. STRETCH: *p* = 0.015; MICT vs. STRETCH: *p* = 0.011), but there was no significant difference between HIIT and MICT (*p* = 0.95). There were no significant group differences at baseline with respect to age, PSQI scores (global sleep quality, sleep efficiency, sleep duration, and components one through seven), BMI, PSS, or MoCA (all *p* > 0.050).

**Table 1:** Pre and post outcome measures for the high-intensity interval training (HIIT), moderate-intensity continuous training (MICT), and stretching (STRETCH) groups.

	HIIT (*n* = 20)		MICT (*n* = 19)		STRETCH (*n* = 22)	
Age	72.4 (4.5)		72.3 (6.2)		71.1 (6.5)	
Sex (% female)	70%		47%		68%	
	Pre	Post	Pre	Post	Pre	Post
Sleep duration (hours)	6.8 (1.2)	6.8 (1.2)	6.5 (1.4)	6.7 (1.5)	7.1 (1.6)	6.8 (1.2)
Sleep efficiency (%)	80.6 (15.9)	79.7 (14.3)	78.6 (15.3)	84.2 (15.4)	73.9 (15.3)	78.5 (15.9)
Global PSQI score	7.0 (3.5)	7.3 (4.6)	7.8 (4.6)	7.6 (4.2)	6.8 (4.6)	6.2 (3.7)
Subjective sleep quality	1.1 (0.8)	1.1 (0.9)	1.3 (0.7)	1.1 (0.8)	0.8 (0.8)	0.9 (0.8)
Sleep latency	1.2 (1.1)	0.9 (0.7)	1.2 (1.1)	0.8 (0.7)	1.3 (1.1)	0.7 (0.6)
Sleep duration	0.8 (0.8)	0.8 (0.8)	1.1 (1.0)	1.0 (0.9)	0.7 (0.9)	0.9 (0.8)
Habitual sleep efficiency	0.9 (1.1)	1.2 (1.3)	1.2 (1.2)	0.8 (1.2)	1.2 (1.3)	1.0 (1.2)
Sleep disturbances	1.6 (0.5)	1.6 (0.6)	1.7 (0.6)	1.6 (0.5)	1.5 (0.6)	1.3 (0.6)
Use of sleeping medication	0.5 (0.9)	0.7 (1.1)	0.8 (1.3)	0.8 (1.3)	0.7 (1.2)	0.6 (1.0)
Daytime dysfunction	1.0 (0.6)	0.9 (0.4)	0.7 (0.7)	0.5 (0.5)	0.7 (0.6)	0.7 (0.5)
Predicted VO_2_ peak (ml/kg/min)	24.8 (6.3)^*^	31.0 (5.6)	24.9 (5.5)^*^	30.7 (4.4)	19.2 (6.9)	18.3 (6.8)
BMI (kg/m^2^)	27.1 (4.0)	27.4 (4.0)	28.1 (3.7)	27.9 (3.5)	29.5 (6.0)	29.7 (6.3)
PSS	13.3 (6.0)	11.8 (5.7)	14.2 (5.8)	12.6 (6.5)	13.6 (5.1)	12.3 (6.2)
MoCA	25.5 (3.1)	26.0 (2.9)	26.1 (3.1)	25.9 (3.2)	26.3 (2.5)	25.5 (2.7)

* Significantly different from STRETCH (*p* < 0.050).

Sex is presented as percentage of females; all other data is presented as mean (SD). ANOVAs revealed a main effect of exercise group on cardiorespiratory fitness (*p* = 0.016), such that HIIT and MICT had significantly higher VO_2_ peak at baseline than STRETCH (HIIT vs. STRETCH: *p* = 0.015; MICT vs. STRETCH: *p* = 0.011), but there was no significant difference between HIIT and MICT (*p* = 0.95). BMI, Body Mass Index; MoCA, Montreal Cognitive Assessment; PSQI, Pittsburgh Sleep Quality Index; PSS, Perceived Stress Scale.

### Exercise Intervention: Group Effects

There was no effect of the exercise intervention on global sleep quality, sleep efficiency, sleep duration, or the seven component scores (all *p* > 0.050). However, the exercise intervention induced the expected cardiorespiratory fitness adaptations [*F*(2, 42) = 13.91, *p* < 0.001, *η_p_*^2^ = 0.40], such that both HIIT and MICT yielded significantly greater improvements in VO_2_ peak than STRETCH (HIIT vs. STRETCH: *p* < 0.001; MICT vs. STRETCH: *p* < 0.001), but there was no significant difference between HIIT and MICT (*p* = 0.48).

### Baseline Characteristics: Subgroup Effects

Using the global measure of sleep quality, participants were categorized as poor (global PSQI score ≥5) or good sleepers (global PSQI score <5; Buysse et al., 1989; Irwin et al., 2008). Table 2 displays baseline characteristics of poor and good sleepers. At baseline, poor sleepers had a significantly lower global sleep quality score than good sleepers [*F*(1, 55) = 53.38, *p* < 0.001, *η_p_*^2^ = 0.49]. Poor sleepers also had significantly shorter sleep duration [hours; *F*(1, 56) = 17.47, *p* < 0.001, *η_p_*^2^ = 0.24], worse sleep efficiency [*F*(1, 50) = 18.79, *p* < 0.001, *η_p_*^2^ = 0.27], and scored higher on six of seven PSQI component scores, including subjective sleep quality [*F*(1, 55) = 34.98, *p* < 0.001, *η_p_*^2^ = 0.39], sleep latency [*F*(1, 56) = 33.72, *p* < 0.001, *η_p_*^2^ = 0.37], sleep duration [*F*(1, 56) = 22.49, *p* < 0.001, *η_p_*^2^ = 0.29], habitual sleep efficiency [*F*(1, 56) = 18.45, *p* < 0.001, *η_p_*^2^ = 0.25], sleep disturbances [*F*(1, 56) = 30.71, *p* < 0.001, *η_p_*^2^ = 0.35], and daytime dysfunction [*F*(1, 56) = 5.39, *p* = 0.024, *η_p_*^2^ = 0.09]. There were no significant differences between poor and good sleepers for use of sleeping medication [*F*(1, 55) = 3.35, *p* = 0.073, *η_p_*^2^ = 0.06], but this trended in the same direction as the other variables. Furthermore, poor sleepers reported significantly higher perceived stress than good sleepers [*F*(1, 56) = 6.07, *p* = 0.017, *η_p_*^2^ = 0.10], but did not differ on VO_2_ peak, BMI, or MoCA (all *p* > 0.050).

**Table 2:** Baseline characteristics for poor and good sleepers.

	Poor sleepers (*n* = 43)	Good sleepers (*n* = 18)
Sleep duration (hours)	6.4 (1.2)^**^	7.9 (1.2)
Sleep efficiency (%)	72.8 (13.8)^**^	91.0 (13.8)
Global PSQI score	9.1 (3.0)^**^	2.6 (3.0)
Subjective sleep quality	1.3 (0.6)^**^	0.3 (0.6)
Sleep latency	1.6 (0.9)^**^	0.2 (0.9)
Sleep duration	1.2 (0.8)^**^	0.1 (0.8)
Habitual sleep efficiency	1.5 (1.1)^**^	0.2 (1.1)
Sleep disturbances	1.8 (0.4)^**^	1.1 (0.4)
Use of sleeping medication	0.8 (1.1)	0.3 (1.1)
Daytime dysfunction	0.9 (0.6)^*^	0.5 (0.6)
Predicted VO_2_ peak (ml/kg/min)	22.1 (6.3)	24.6 (6.3)
BMI (kg/m^2^)	27.9 (4.7)	29.0 (4.8)
PSS	14.8 (5.2)^*^	11.1 (5.3)
MoCA	25.8 (2.8)	26.2 (2.9)

* *p* < 0.050;

** *p* < 0.010.

Data are presented as mean (SD). ANCOVAs revealed that poor sleepers had significantly lower global sleep quality score than good sleepers (*p* < 0.001), as well as significantly shorter sleep duration (hours), worse sleep efficiency, and scored higher on six of seven PSQI component scores, including subjective sleep quality, sleep latency, sleep duration, habitual sleep efficiency, sleep disturbances (all *p* < 0.001), and daytime dysfunction (*p* = 0.024). Poor sleepers also reported significantly higher perceived stress than good sleepers (*p* = 0.017), but did not differ on use of sleeping medication, VO_2_ peak, BMI, or MoCA (all *p* > 0.050). Sleep variables are adjusted for age, sex, and BMI. Predicted VO_2_ peak, BMI, PSS, and MoCA are adjusted for age and sex. BMI, Body Mass Index; MoCA, Montreal Cognitive Assessment; PSQI, Pittsburgh Sleep Quality Index; PSS, Perceived Stress Scale.

### Exercise Intervention: Subgroup Effects

**Figure 2:**
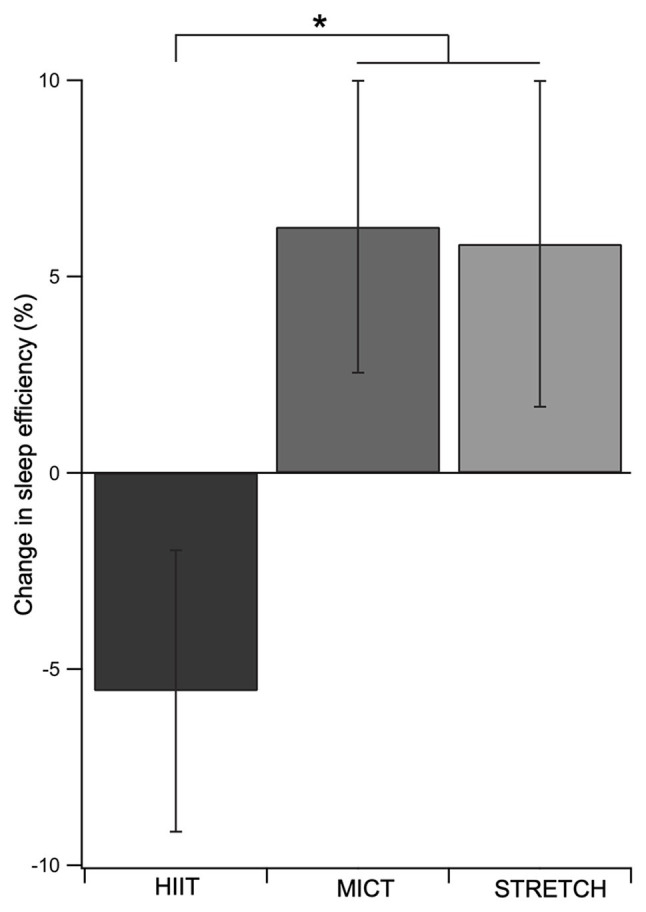
Change in sleep efficiency as a function of exercise group in poor sleepers. Both MICT and STRETCH groups experienced greater improvements in sleep efficiency than the HIIT group. This was supported by an ANCOVA, which revealed a main effect of group for sleep efficiency. Age, sex, and body mass index were included as covariates. Error bars represent SEM for each group. ^*^p ≤ 0.05.

The effects of the exercise interventions on poor sleepers are presented in Table 3. The exercise intervention improved sleep efficiency for poor sleepers, except for when done at a high intensity (Figure 2). This was supported by a main effect of group [*F*(2, 29) = 3.27, *p* = 0.053, *η_p_*^2^ = 0.18], whereby MICT and STRETCH resulted in improvements in sleep efficiency, but HIIT did not (MICT vs. HIIT: *p* = 0.030; STRETCH vs. HIIT: *p* = 0.050). MICT and STRETCH groups were not statistically different (*p* = 0.94). No other sleep measure was significantly affected by the intervention (all *p* > 0.050).

**Table 3:** Pre and post sleep measures for the HIIT, MICT, and STRETCH groups in poor sleepers only.

	HIIT (*n* = 15)		MICT (*n* = 15)		STRETCH (*n* = 13)	
	Pre	Post	Pre	Post	Pre	Post
Sleep duration (hours)	6.7 (1.3)	6.5 (1.2)	6.1 (1.2)	6.5 (1.5)	6.3 (1.5)	6.3 (1.3)
Sleep efficiency (%)	77.6 (16.7)	75.2 (13.7)	73.4 (13.0)	80.8 (15.1)	66.6 (13.5)	74.1 (17.3)
Global PSQI score	8.3 (2.8)	9.1 (3.9)	9.5 (3.8)	8.6 (4.0)	9.6 (4.0)	8.3 (3.6)
Subjective sleep quality	1.3 (0.7)	1.4 (0.8)	1.5 (0.6)	1.3 (0.7)	1.2 (0.7)	1.3 (0.8)
Sleep latency	1.5 (1.1)	1.1 (0.6)	1.5 (1.1)	1.0 (0.7)	1.9 (0.9)	1.0 (0.4)
Sleep duration	0.9 (0.8)	1.0 (0.8)	1.4 (0.9)	1.1 (1.0)	1.2 (0.8)	1.3 (0.8)
Habitual sleep efficiency	1.1 (1.2)	1.6 (1.2)	1.5 (1.2)	1.0 (1.2)	1.9 (1.3)	1.5 (1.3)
Sleep disturbances	1.8 (0.4)	1.7 (0.6)	1.9 (0.5)	1.7 (0.5)	1.8 (0.6)	1.6 (0.5)
Use of sleeping medication	0.7 (1.0)	0.9 (1.2)	1.0 (1.4)	1.0 (1.4)	0.8 (1.3)	0.5 (1.0)
Daytime dysfunction	1.1 (0.7)	0.9 (0.5)	0.8 (0.7)	0.6 (0.5)	0.8 (0.6)	0.8 (0.4)

Data is presented as mean (SD). PSQI, Pittsburgh Sleep Quality Index.

### Cardiorespiratory Fitness and Sleep

There was no correlation between change in cardiorespiratory fitness and change in sleep efficiency in poor sleepers [*r*(22) = −0.05, *p* = 0.81].

## Discussion

The present community-based study examined the effect of exercise intensity on sleep quality in older adults. The exercise intervention had no effect on sleep when examining the entire sample of older adults. However, subgroup analyses revealed that among poor sleepers, both MICT and STRETCH improved sleep efficiency, whereas HIIT did not.

The majority of the community-dwelling older adults in our sample were classified as poor sleepers. These poor sleepers were significantly impaired across all PSQI components except for the use of sleeping medication, whereby neither group were heavily prescribed. These results highlight the prevalence and extent of sleep impairments among older adults in the community. The low endorsement of sleep medication by poor sleepers may signify a lack of medical support for their impairment. If untreated, impaired sleep can disrupt many aspects of health and accelerate age-related decline (Foley et al., 1999; Dew et al., 2003; Reid et al., 2006). Indeed, our poor sleepers also had higher levels of psychological stress than good sleepers. Although the present results cannot speak to whether this was a cause or consequence of poor sleep, prior research suggests it can be both; poor sleep creates more psychological stress, which in turn can further shorten sleep duration and impair sleep quality (Åkerstedt, 2006). Unfortunately, this creates a negative feedback loop to perpetuate sleep impairments. Within this context, the results point to a critical need for effective interventions to help older adults manage sleep.

Our results support the accumulating evidence that exercise in the form of MICT improves sleep in older adults (King et al., 1997, 2008; Tworoger et al., 2003; Passos et al., 2011). However, this was only seen among those with poor sleep quality. Indeed, it has been suggested that good sleepers are unlikely to exhibit large improvements in sleep following an exercise intervention due to ceiling effects (Youngstedt, 2003, 2005), providing a potential explanation for why we did not find an effect of exercise on sleep when examining all older adults (i.e., both good and poor sleepers). The novel contribution here was contrasting MICT with HIIT – a popular alternative form of aerobic exercise. At the group level, HIIT was not effective at improving sleep efficiency in poor sleepers, while MICT was. These findings suggest there may be an intensity threshold for the sleep-promoting effects of exercise, such that exercise at a high intensity could be harmful to sleep. Indeed, high-intensity exercise may elicit heightened physiological arousal and muscle soreness, counteracting the potential beneficial effects of exercise on sleep and tipping the balance toward inhibiting, rather than promoting, sleep (Borbély, 1982; Driver and Taylor, 2000; Fuller et al., 2006).

Despite the potential for exercise to promote sleep, the underlying mechanisms are not fully understood (Uchida et al., 2012; Kredlow et al., 2015). One proposed mechanism is that exercise-induced improvements in cardiorespiratory fitness promote sleep (Shapiro et al., 1984). Our results do not lend support for this proposed mechanism. Instead, we found no relationship between change in fitness and change in sleep efficiency among the poor sleepers. Furthermore, sleep efficiency improved in the STRETCH group despite no change in cardiorespiratory fitness. These findings suggest the sleep-promoting effects of exercise may be driven by changes in other physiological or psychological variables, such as increased energy consumption and metabolic rate (Morselli et al., 2012) or changes in mood (Buman and King, 2010). Future research is needed to elucidate the underlying mechanisms through which exercise improves sleep in older adults to help further refine implementation.

While this study makes important contributions to our understanding of the relationship between exercise intensity and sleep quality in older adults, it is not without its limitations. Firstly, this was a community-based study with a focus on feasibility and implementation, and as a result did not meet all requirements for a clinical trial. Secondly, the STRETCH group in this study was originally designed to control for the social factors associated with participating in an exercise intervention that are known to affect cognition (the main focus of the original study; Kovacevic et al., 2019). However, stretching improves sleep (Figure 2), possibly through physical relaxation (King et al., 1997), and therefore is not the ideal control condition for sleep outcomes. Thus, we treated the STRETCH group as an additional treatment group and did not have a control group. Follow up studies should incorporate a non-active control condition, such as a health education program, to isolate the effects of exercise intensity on sleep. Thirdly, while the MICT and HIIT groups were matched for total work output, the duration of exercise differed between the MICT, HIIT, and STRETCH groups, which may have influenced sleep quality as well. Finally, because sleep quality was not the primary outcome for the original intervention, there were too few good sleepers to examine the effect of the exercise intervention. While poor sleepers are the target group for treatment, future studies should aim to include a larger sample size of both poor and good sleepers for a full comparison.

Overall, the findings from this study suggest that both MICT and stretching may be more effective than HIIT for improving sleep in older adults with poor sleep quality. Critically, this is the first study to investigate the impact of HIIT vs. MICT on sleep in older adults. These results help inform an optimal exercise prescription for improving sleep quality to help keep older adults healthier longer.

## Data Availability Statement

The raw data supporting the conclusions of this article will be made available by the authors, without undue reservation.

## Ethics Statement

The studies involving human participants were reviewed and approved by Hamilton Integrated Research Ethics Board, McMaster University. The patients/participants provided their written informed consent to participate in this study.

## Author Contributions

AB and JJH: data analysis, manuscript writing. AK: data collection, manuscript editing. TK: data analysis, manuscript editing. All authors contributed to the article and approved the submitted version.

### Conflict of Interest

The authors declare that the research was conducted in the absence of any commercial or financial relationships that could be construed as a potential conflict of interest.
